# Cultural effects on the association between election outcomes and face-based trait inferences

**DOI:** 10.1371/journal.pone.0180837

**Published:** 2017-07-10

**Authors:** Chujun Lin, Ralph Adolphs, R. Michael Alvarez

**Affiliations:** 1 Division of Humanities and Social Sciences, California Institute of Technology, Pasadena, California, United States of America; 2 Division of Biology and Biological Engineering, California Institute of Technology, Pasadena, California, United States of America; Harvard Medical School, UNITED STATES

## Abstract

How competent a politician looks, as assessed in the laboratory, is correlated with whether the politician wins in real elections. This finding has led many to investigate whether the association between candidate appearances and election outcomes transcends cultures. However, these studies have largely focused on European countries and Caucasian candidates. To the best of our knowledge, there are only four cross-cultural studies that have directly investigated how face-based trait inferences correlate with election outcomes across Caucasian and Asian cultures. These prior studies have provided some initial evidence regarding cultural differences, but methodological problems and inconsistent findings have complicated our understanding of how culture mediates the effects of candidate appearances on election outcomes. Additionally, these four past studies have focused on positive traits, with a relative neglect of negative traits, resulting in an incomplete picture of how culture may impact a broader range of trait inferences. To study Caucasian-Asian cultural effects with a more balanced experimental design, and to explore a more complete profile of traits, here we compared how Caucasian and Korean participants’ inferences of positive and negative traits correlated with U.S. and Korean election outcomes. Contrary to previous reports, we found that inferences of competence (made by participants from both cultures) correlated with both U.S. and Korean election outcomes. Inferences of open-mindedness and threat, two traits neglected in previous cross-cultural studies, were correlated with Korean but not U.S. election outcomes. This differential effect was found in trait judgments made by both Caucasian and Korean participants. Interestingly, the faster the participants made face-based trait inferences, the more strongly those inferences were correlated with real election outcomes. These findings provide new insights into cultural effects and the difficult question of causality underlying the association between facial inferences and election outcomes. We also discuss the implications for political science and cognitive psychology.

## Introduction

Numerous studies have reported that trait inferences made by participants who had no previous knowledge of the political candidates, and who looked at the candidates’ photos for as briefly as 100 milliseconds, correlate with real election outcomes [1–4]. This was initially studied for Australian elections [5] and was made most popular by the later studies for U.S. elections [1–3]. Subsequent research has examined how these face-based trait evaluations might be associated with election outcomes in other countries than the U.S. Supportive evidence reinforcing the original results has been found in Britain [6–9], Germany [10–11], France [12], Finland [13] (but see [14]), Ireland [15], Switzerland [16], Bulgaria [17], Denmark [18], Italy [19], Australia [9, 20], New Zealand [9], Brazil [21], Mexico [21], Japan [22], China [23], and Taiwan (ROC) [24, 25] (but see [26] for insignificant effects found in South Korea). Facial inferences made by both human subjects (cf. citations above) and computer algorithms [27] have been demonstrated to associate with election outcomes across cultures. Additional studies have extended this literature by exploring the association between election outcomes and a broader range of facial attributions, such as smile intensity in photos [28], and facial cues that reveal candidates’ political affiliations [29, 30] and personality (e.g., extraverted/enthusiastic and disorganized/careless) [3]. Our present study focused on direct comparisons between Caucasian and Asian cultures, and on traits that are closely related to the initial study [5] (e.g., competence).

Understanding cultural effects advances our knowledge about how candidate appearances associate with election outcomes, which could have complex explanations. For instance, one study [28] has found that American politicians show more excited smiles in their official photos than do Chinese/Taiwanese politicians. Cultural nuances such as this might mediate how face-based trait inferences correlate with real election outcomes because the inferences of traits are influenced by the perceptions of emotional expressions of the faces [31–35]. Differences in what traits are valued across cultures [36, 37] might be yet another contributing factor.

Although there are numerous studies of this topic across a range of cultures (cf. citations above), few have directly compared Caucasian and Asian cultures in the same study. To the best of our knowledge, there are only four cross-cultural studies that have directly investigated how face-based trait inferences made by human subjects correlate with real election outcomes, and made explicit comparisons between Caucasian and Asian cultures [22, 24–26]. One of these studies [22] found that inferences of power traits (dominance and facial maturity) correlated with U.S. but not Japanese election outcomes, while inferences of warmth traits (likeability and trustworthiness) correlated with Japanese but not U.S. election outcomes. Such different trait-election associations were observed for inferences made by both Caucasian and Asian participants. However, while the stimuli for U.S. candidates used in this study were winners and runner-ups in matched electoral races, the stimuli for Japanese candidates were not matched (winners and losers were from different electoral races). Another of these studies [26] found that inferences of competence correlated much more strongly with U.S. election outcomes than with Korean election outcomes. The candidates who were perceived as more competent by their Korean participants won in 61.92% of the U.S. elections but in only 49.98% of the Korean elections (which was below chance). The candidates who were perceived as more competent by their U.S. participants won in 60.31% and 52.85% of the electoral races in the U.S. and Korea, respectively. However, this study [26] counterbalanced the ordering of image groups only for Caucasian participants but not for Korean participants; thus, all Korean participants evaluated U.S. candidates first, introducing possibly confounding order effects into the study.

Caucasian-Asian cultural differences were also studied by [24, 25], which compared U.S. and Taiwan elections. Unlike [22], [24] found that inferences of trustworthiness (one of the two domains of warmth traits) made by Caucasian participants were correlated with neither U.S. nor Taiwan election outcomes, while those made by Asian participants were negatively correlated with U.S. election outcomes. Counter to the argument in [26] that trait inferences were less important in Asian cultures and should be less associated with Asian elections, [24, 25] showed that inferences of some traits (e.g., social competence) correlated even more strongly with Taiwan than U.S. election outcomes. While [26] was a within-subject design, [22, 24–25] were not: participants in the latter two studies evaluated only ten pairs of faces from each culture, randomly chosen from the image pool. The discrepancies in the findings among these four studies, and the unbalanced experimental designs they used, complicate our understanding of how Caucasian and Asian cultural effects might mediate the association between appearance-based trait inferences and real election outcomes. To help clarify this issue was one motivation of our present study.

In addition to the lack of consensus on cultural effects (of both participants and election locations) as reviewed above, there is a second aspect of this topic that remains under-investigated: negative facial cues. While a few studies with Caucasian politicians have found that negative traits inferred from faces are strongly associated with election outcomes [3, 38–40], more attention has been given to investigating positive traits such as warmth, competence, trustworthiness and dominance [41–44], which tend to be strongly inter-correlated. All the four Caucasian-Asian cross-cultural studies above [22, 24–26] examined only positive traits. This gap in the study of how negative traits might influence voter decisions is important because positive and negative traits could influence voter decisions through distinct mechanisms [38]. Negative advertising has been employed in political campaigns for decades. Lyndon Johnson’s landslide victory in the 1964 U.S. presidential election is believed to owe much to the “Daisy” advertisement which attacked his opponent Barry Goldwater for being militarily aggressive. In the 2016 presidential election campaigns, Donald Trump questioned the intelligence of his rival Jeb Bush, and Republican attack-advertisements portrayed Hillary Clinton as a liar. Such anecdotal evidence, together with findings in [3, 38–40] suggest that it is important to understand how inferences of a variety of negative traits influence voting, and how culture mediates these effects. A second motivation for our present study was thus to provide a more comprehensive investigation of both positive and negative traits in a cross-cultural context.

To provide a more balanced experimental design (in participants, stimuli, and procedures) for studying cross-cultural effects, and to investigate multiple positive and negative traits, we asked Caucasian and Korean participants to make inferences of competence, open-mindedness, threat, and corruption for pairs of real political candidates from past U.S. and Korean elections. We found that the traits that were most strongly associated with election outcomes differed between U.S. and Korean elections, but that the associations were consistent across both Caucasian and Korean participants. These results provide new insights into the difficult question of causality underlying the association between face-based trait inferences and election outcomes. They also have implications for studies of political behavior: they suggest that it is important to include both candidate traits and their cultural backgrounds in the classic vote choice model.

## Materials and methods

### Participants

Caucasian participants (N = 40; 20 male; Age (M = 31, SD = 6.9)) and Korean participants (N = 40; 20 male; Age (M = 29, SD = 6.4)) with normal (or corrected-to-normal) vision were recruited from the general population in Southern California in early 2016. All Caucasian participants self-reported as “White, non-Hispanic” in the prescreening survey. All Korean participants were recruited through Korean-language advertisements. To balance the two subject pools, we recruited both Caucasians and Koreans from nearby colleges, churches, and through similar websites (e.g., Craigslist and Reddit for Caucasians, and Radiokorea for Koreans) (S1 Table). Based on earlier work by [1] (SOM), we established that a sample size of forty participants from each cultural background would be necessary; their study showed that the average individual accuracy of face-based competence inferences predicting U.S. election outcomes increased substantially as the sample size approached 40 participants, but that the benefit of additional participants diminished after that point.

At the time of the experiments, our Caucasian participants had been in the U.S. for an average of 30 years (SD = 8.5, median = 30). Among the forty Korean participants, thirty-two of them were born in South Korea and had lived in South Korea for an average of 19 years (SD = 10.02, median = 19); three were born in China, Canada, and Germany respectively and had lived in South Korea for an average of 11 years; and the other five were born in the U.S. Twenty-three of our Korean participants spoke only Korean at home, fifteen of them spoke both Korean and English at home, and the other two spoke only English at home. All procedures were carried out in compliance with the approval of the Caltech Institutional Review Board. All participants signed a written informed consent before the study and received between $15 to $40 (depending on their travel distance) for their participation in the study. All participants completed all parts of the study and none was excluded from the analysis.

### Stimuli

Stimuli were headshot photographs of real political candidates who ran in U.S Congressional elections, or in Korean Assembly elections. For Caucasian candidates, following the procedure in [26], we used a randomly selected set of 45 pairs of candidates (4 female pairs) from a previously establish database [1–3] (http://tlab.princeton.edu/databases/politicians). For Korean candidates, we used the same 45 pairs of candidates (2 female pairs) as in [26]. Images were paired according to actual electoral races, with one being the winner and the other the runner-up. Only electoral races in which candidates were of the same sex and ethnicity were included. Any conspicuous background such as the capital or a national flag was removed and replaced with a gray background. All images were in black-and-white, of similar clarity, with frontal facing and centrally presented smiling faces, and were cropped to similar sizes according to the intraocular distance. When presented on the computer screen, all images had a standard size of 3.2 cm (width) x 4.5 cm (height) [1]. All materials can be accessed at https://osf.io/qx54t/?view_only=f504dcb528aa4546a2b01ee9e54f72b3.

### Procedure

**Fig 1:**
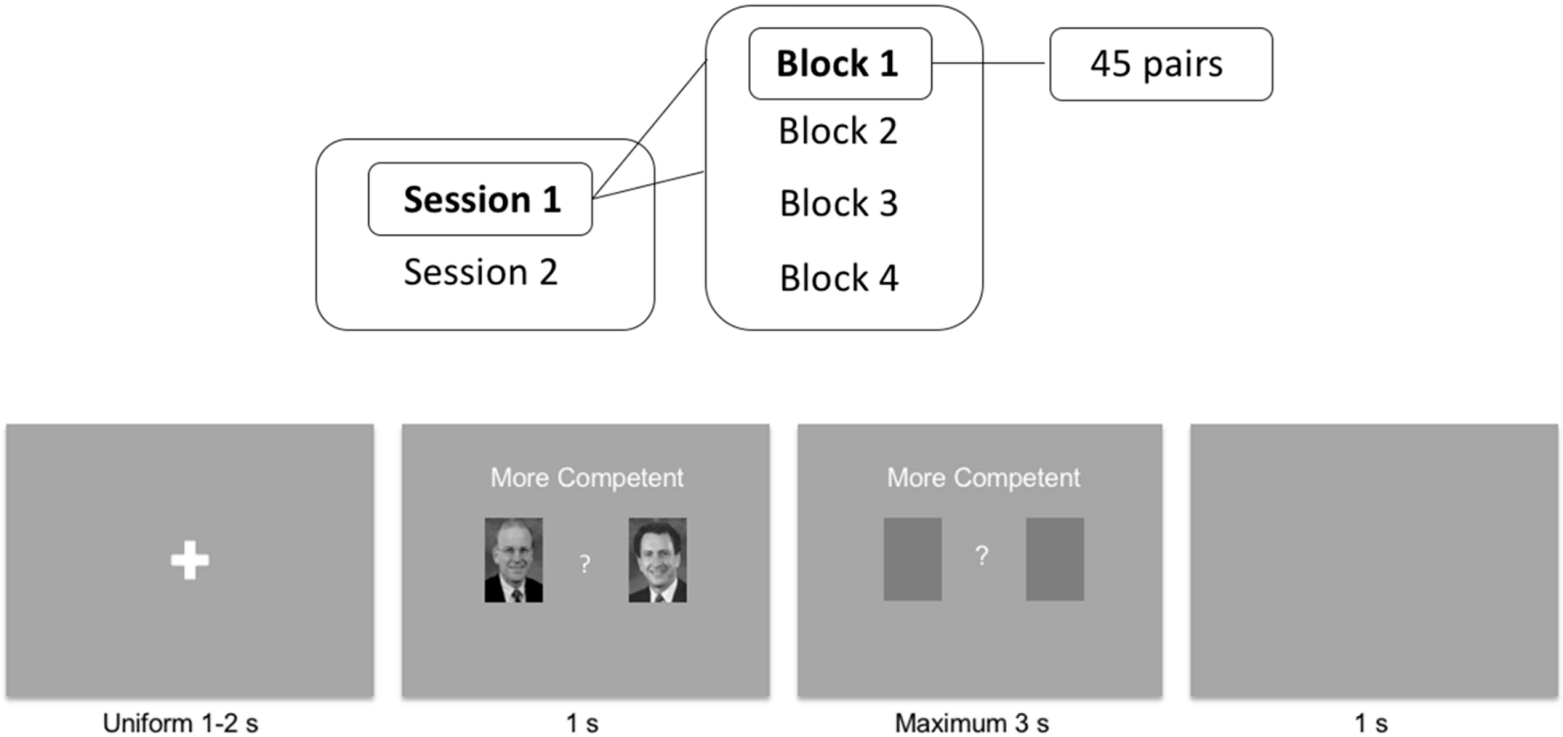
Experiment procedure and image display screens. (a) The schematic diagram of the full experiment. (b) Example of image display screens in the competence evaluation block. At the beginning of each block, there were instructions on the screen indicating which trait the participant was asked to evaluate. Then for each pair of candidates, participants first focused on the cross of the fixation screen which lasted for 1–2 seconds; then the images of the pair of candidates were up for 1 second; participants could make a decision as soon as the images appeared; after the images disappeared, participants had a maximum of 3 seconds to enter their choice. As soon as a valid key was pressed (i.e., press “A” if their choice was the candidate on the left and press “L” if their choice was the candidate on the right), a grey screen was up for a 1 second inter-stimulus interval.

All experiments were carried out at the same laboratory at Caltech with the same experimenter. Participants completed two sessions of ratings on a computer: one for Caucasian candidates and the other for Korean candidates (Fig 1a). The ordering of the two sessions was counterbalanced across participants, for both Caucasian and Korean participants. In each session, there were four blocks, each corresponding to one of the four traits: competence, open-mindedness, threat, and corruption (Fig 1a). The ordering of the four blocks was randomized for each participant. The questions on competence and threat were worded as in [38]; those on open-mindedness and corruption were worded in the same way as the competence question. In each block, participants viewed images of the 45 pairs of the political candidates (Fig 1a), and for each pair of candidates they indicated which candidate was their choice for that trait (e.g., which candidate in a pair looked more competent to hold national congressional office) (Fig 1b). The ordering of the 45 pairs of images was randomized for each participant in each block. Positions of the images were randomized in each block and counterbalanced across blocks for each participant: in each block, for half of the races the winners were positioned on the right-hand side and for the other half they were positioned on the left-hand side; the winner of a pair appeared on one side in two of the blocks (first and third blocks) and the other side in the other two blocks.

After completing each session, participants were asked whether they recognized any of the candidates. If a participant recognized any of the candidates in a pair, his/her responses for this pair of candidates were excluded from further analysis. After completing both sessions, participants completed a paper-and-pencil survey on demographic characteristics, values, and political attitudes. All data files and analysis codes can be accessed at https://osf.io/qx54t/?view_only=f504dcb528aa4546a2b01ee9e54f72b3.

## Results

### Reliability of face-based trait inferences across subjects

First, we determined the reliability of our participants’ trait inferences. For each trait, we calculated the intraclass correlation coefficients (ICCs) with responses from all participants (across both cultures) for U.S. and Korean candidates respectively (using the R function ICC (type = ‘ICC2k’)). As expected, across all traits and for both cultures of candidates, the ICCs were high, ranging from 0.78 to 0.87 (all p-value < 0.001), similar to those reported in [26]. These results implied that most of the variance in our participants’ candidate choices was explained by the variance across the candidate pairs instead of the variance among participants. Thus, in line with previous reports [21–22, 26, 45–46], we found high consensus on face-based trait inferences across participants from both cultures.

### Consistency in face-based inferences across traits

Next, we determined the degree to which our participants made consistent inferences of traits. Given the high consensus on face-based trait inferences across participants, we analyzed the consistency of trait inferences at the aggregate level. For each pair of faces, we calculated for the winning candidate the percentages of participants (including both Caucasian and Korean participants) who decided he/she was their choice for being more competent, more open-minded, more threatening, and more corrupt. Using these percentages as the dependent measures, we calculated Spearman correlations between inferences on each pair of traits. We found strong positive correlations between inferences of traits with the same valence (positive or negative) and strong negative correlations between those with opposite valences (Table 1). These results suggested that, at the aggregate level, our participants made consistent trait inferences for both Caucasian and Korean candidates. It is noteworthy that the correlations we observed were nearly identical in magnitude for the evaluations of Caucasian candidates and Korean candidates. Interestingly, both perceived threat and corruption were more strongly correlated with perceived open-mindedness than perceived competence.

**Table 1:** Spearman correlations between aggregate inferences of different traits.

	Evaluations of Caucasian Candidates			Evaluations of Korean Candidates		
	Competence	O	T	Competence	O	T
Open-mindedness (O)	0.62			0.63		
	[0.39, 0.79]			[0.40, 0.79]		
Threat (T)	-0.60	-0.72		-0.58	-0.66	
	[-0.77, -0.33]	[-0.85, -0.49]		[-0.74, -0.35]	[-0.81, -0.46]	
Corruption	-0.54	-0.63	0.69	-0.60	-0.63	0.85
	[-0.74, -0.22]	[-0.76, -0.41]	[0.44, 0.83]	[-0.75, -0.37]	[-0.78, -0.39]	[0.71, 0.93]

All p-value < 0.001. 95% Confidence Intervals were presented in [].

### Associations between face-based trait inferences and election outcomes in the U.S. and Korea

**Fig 2:**
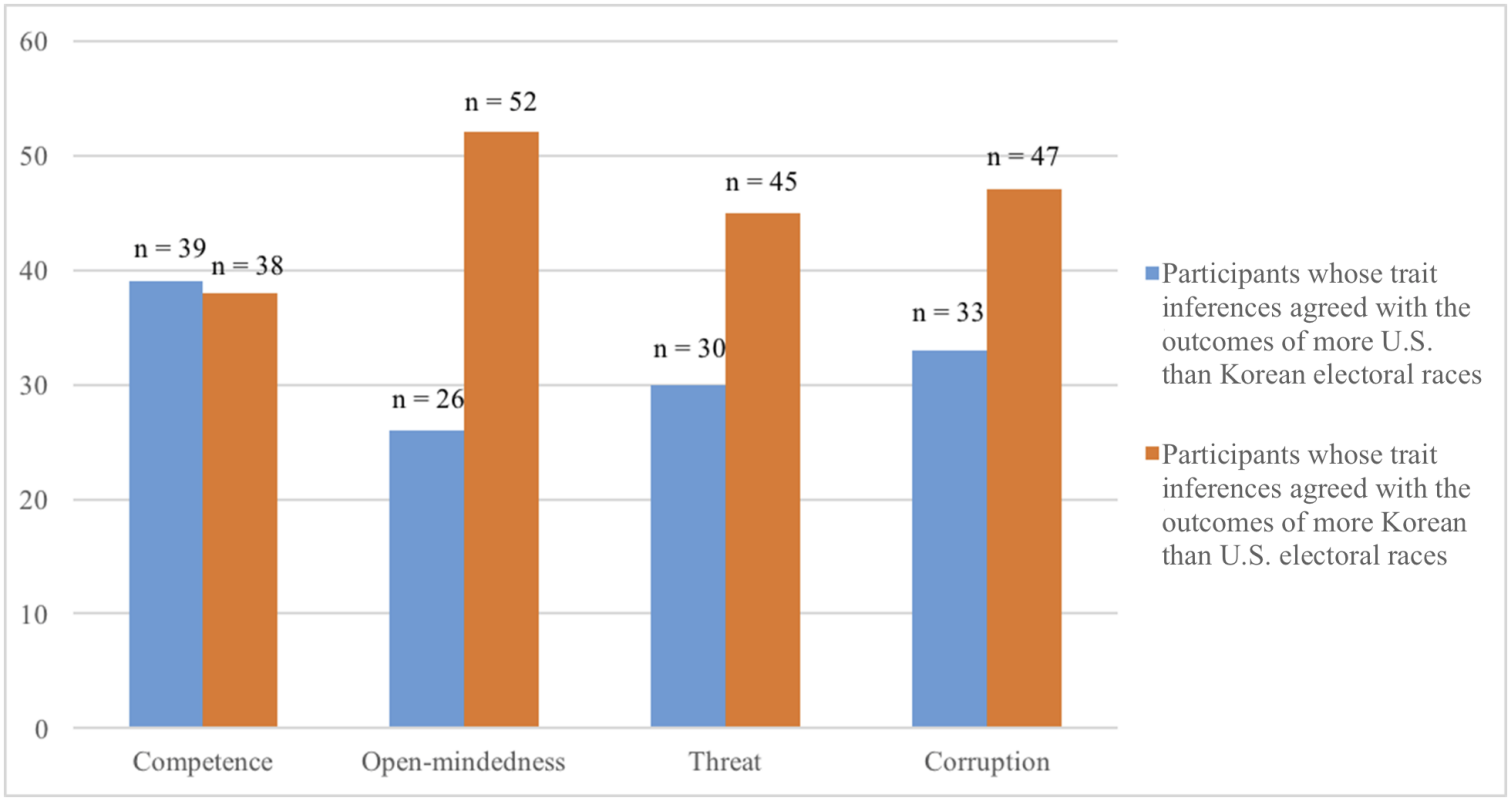
The number of participants whose trait inferences agreed with the outcomes of more electoral races in one country than the other. The blue histogram represents the numbers of participants whose trait inferences agreed with the outcomes of more U.S. than Korean elections. The orange histogram represents the numbers of participants whose trait inferences agreed with the outcomes of more Korean than U.S. elections. For brevity, the category U.S. = Korean was omitted from the graph. All participants (N = 80).

Our main aim was to investigate whether face-based inferences about a range of traits about candidates were associated with which candidates won or lost in U.S. and Korean elections. We thus compared our participants’ face-based trait inferences against real election outcomes. First, we looked at the data at the individual level. For each participant, we calculated the percentages of electoral races in which the candidate who was perceived as more competent, more open-minded, less threatening, and less corrupt, won the race. (Associations such as these percentages are often called “predictions” in the literature [1] even though they are fundamentally correlational and not causal in nature; to avoid confusion, we will generally use the terms “correlation” or “association”.) Then for each trait, we calculated the number of participants whose inferences agreed with the outcomes of more U.S. than Korean electoral races, and the number of participants whose inferences agreed with the outcomes of more Korean than U.S. electoral races (Fig 2). We found that the agreement between competence inferences and election outcomes were similar for U.S. and Korean elections. On the other hand, for the majority of the participants, their inferences of open-mindedness, threat, and corruption agreed with the outcomes of more Korean electoral races than U.S. electoral races.

Next, we looked at the data at the group level. We averaged these percentages of agreement over all participants (N = 80), Caucasian participants (N = 40), and Korean participants (N = 40). To see whether the agreement between inferences of a trait and election outcomes was better than chance, we performed one-sided t-tests on the percentages of agreement against 50%. To see whether the association between trait inferences and election outcomes was stronger in one country than the other, we performed two-sided t-tests on the percentages of agreement across the two countries.

#### Competence

Candidates who were perceived as more competent by our participants in the lab won in more than 50% of the electoral races in both the U.S. and Korea (Table 2, columns a and b). We reproduced the results reported in the initial study [1] that Caucasian participants’ judgments of competence were associated with winners in U.S. elections. Though [1] recruited college students as participants and our participants were from the general public, the average percentage of agreement we found was similar to those reported in [1] (SOM): (M = 59%, SD = 7%) for 2000 and 2002 U.S. Senate races and (M = 53%, SD = 10%) for 2004 races.

**Table 2:** Associations between real election outcomes and face-based inferences of competence.

	Average Agreement		Cross-country Comparison
	U.S. Election^a^	Korean Election^b^	U.S—Korean^c^
All participants (N = 80)	**54.60%**	**54.15%**	0.45%
SD	8.46%	7.50%	t (79) = 0.38
95% CI	[53.03%, Inf)	[52.76%, Inf)	[-1.89%, 2.79%]
Caucasian participants (N = 40)	**55.33%**	**55.46%**	-0.13%
SD	8.87%	7.45%	t (39) = -0.08
95% CI	[52.97%, Inf)	[53.47%, Inf)	[-3.38%, 3.14%]
Korean participants (N = 40)	**53.87%**	**52.85%**	1.02%
SD	8.08%	7.41%	t (39) = 0.59
95% CI	[51.71%, Inf)	[50.88%, Inf)	[-2.48%, 4.51%]

^a^Average agreement between U.S. election outcomes and face-based inferences of competence, and its one-sided t-test against chance level 50%.

^b^Average agreement between Korean election outcomes and face-based inferences of competence, and its one-sided t-test against chance level 50%.

^c^Two-sided t-tests on the average agreement across U.S. and Korean elections.

Perceived competence was associated with the outcomes of similar percentages of electoral races in the U.S. and Korea. Two-sided t-tests showed no significant difference in how well perceived competence was associated with the winning candidates in U.S. and Korean elections (Table 2, column c).

#### Open-mindedness

Candidates who were perceived as more open-minded by our participants in the lab won in more than 50% of the Korean electoral races, but this association was not significant for U.S. elections (Table 3, columns a and b). Perceived open-mindedness correlated with Korean election outcomes more strongly than U.S. election outcomes (Table 3, column c).

**Table 3:** Associations between real election outcomes and face-based inferences of open-mindedness.

	Average Agreement		Cross-country Comparison
	U.S. Election^a^	Korean Election^b^	U.S—Korean^c^
All participants (N = 80)	49.47%	**55.46%**	**-5.99%**
SD	8.53%	9.47%	t (79) = -3.99
95% CI	[47.89%, Inf)	[53.70%, Inf)	[-8.98%, -3.00%]
Caucasian participants (N = 40)	49.96%	**56.72%**	**-6.76%**
SD	9.41%	9.99%	t (39) = -2.81
95% CI	[47.45%, Inf)	[54.06%, Inf)	[-11.62%, -1.90%]
Korean participants (N = 40)	48.99%	**54.21%**	**-5.22%**
SD	7.63%	8.87%	t (39) = -2.86
95% CI	[46.95%, Inf)	[51.85%, Inf)	[-8.92%, -1.53%]

^a^Average agreement between U.S. election outcomes and face-based inferences of open-mindedness, and its one-sided t-test against chance level 50%.

^b^Average agreement between Korean election outcomes and face-based inferences of open-mindedness, and its one-sided t-test against chance level 50%.

^c^Two-sided t-tests on the average agreement across U.S. and Korean elections.

#### Threat

Candidates who were perceived as more threatening by our participants in the lab lost in more than 50% of the electoral races in both the U.S. and Korea, but these associations were statistically significant for only Korean elections, and not U.S. elections (Table 4, columns a and b). The average agreement (averaged over all participants and Caucasian participants) for U.S. elections significantly differed from that for Korean elections (Table 4, column c).

**Table 4:** Associations between real election outcomes and face-based inferences of threat.

	Average Agreement		Cross-country Comparison
	U.S. Election^a^	Korean Election^b^	U.S—Korean^c^
All participants (N = 80)	51.50%	**54.43%**	**-2.93%**
SD	7.89%	7.38%	t (79) = -2.33
95% CI	[50.03%, Inf)	[53.05%, Inf)	[-5.43%, -0.43%]
Caucasian participants (N = 40)	51.09%	**55.89%**	**-4.80%**
SD	7.92%	7.56%	t (39) = -2.51
95% CI	[48.98%, Inf)	[53.87%, Inf)	[-8.67%, -0.93%]
Korean participants (N = 40)	51.91%	**52.97%**	-1.06%
SD	7.93%	6.97%	t (39) = -0.66
95% CI	[49.80%, Inf)	[51.11%, Inf)	[-4.29%, 2.17%]

^a^Average agreement between U.S. election outcomes and face-based inferences of threat, and its one-sided t-test against chance level 50%.

^b^Average agreement between Korean election outcomes and face-based inferences of threat, and its one-sided t-test against chance level 50%.

^c^Two-sided t-tests on the average agreement across U.S. and Korean elections.

#### Corruption

Candidates who were perceived as more corrupt by our participants in the lab lost in more than 50% of the electoral races in Korea, but this association was not significant for U.S. elections (Table 5, columns a and b). The average agreement (averaged over all participants and Caucasian participants) for U.S. elections significantly differed from that for Korean elections (Table 5, column c).

**Table 5:** Associations between real election outcomes and face-based inferences of corruption.

	Average Agreement		Cross-country Comparison
	U.S. Election^a^	Korean Election^b^	U.S—Korean^c^
All participants (N = 80)	49.18%	**52.21%**	**-3.03%**
SD	9.47%	8.43%	t (79) = -2.07
95% CI	[47.42%, Inf)	[50.64%, Inf)	[-5.94%, -0.11%]
Caucasian participants (N = 40)	47.50%	**52.46%**	**-4.96%**
SD	10.28%	8.72%	t (39) = -2.29
95% CI	[44.76%, Inf)	[50.14%, Inf)	[-9.33%, -0.59%]
Korean participants (N = 40)	50.86%	51.95%	-1.09%
SD	8.38%	8.24%	t (39) = -0.56
95% CI	[48.62%, Inf)	[49.76%, Inf)	[-5.05%, 2.86%]

^a^Average agreement between U.S. election outcomes and face-based inferences of corruption, and its one-sided t-test against chance level 50%.

^b^Average agreement between Korean election outcomes and face-based inferences of corruption, and its one-sided t-test against chance level 50%.

^c^Two-sided t-tests on the average agreement across U.S. and Korean elections.

### Response-time mediates the associations between face-based trait inferences and real election outcomes

**Fig 3:**
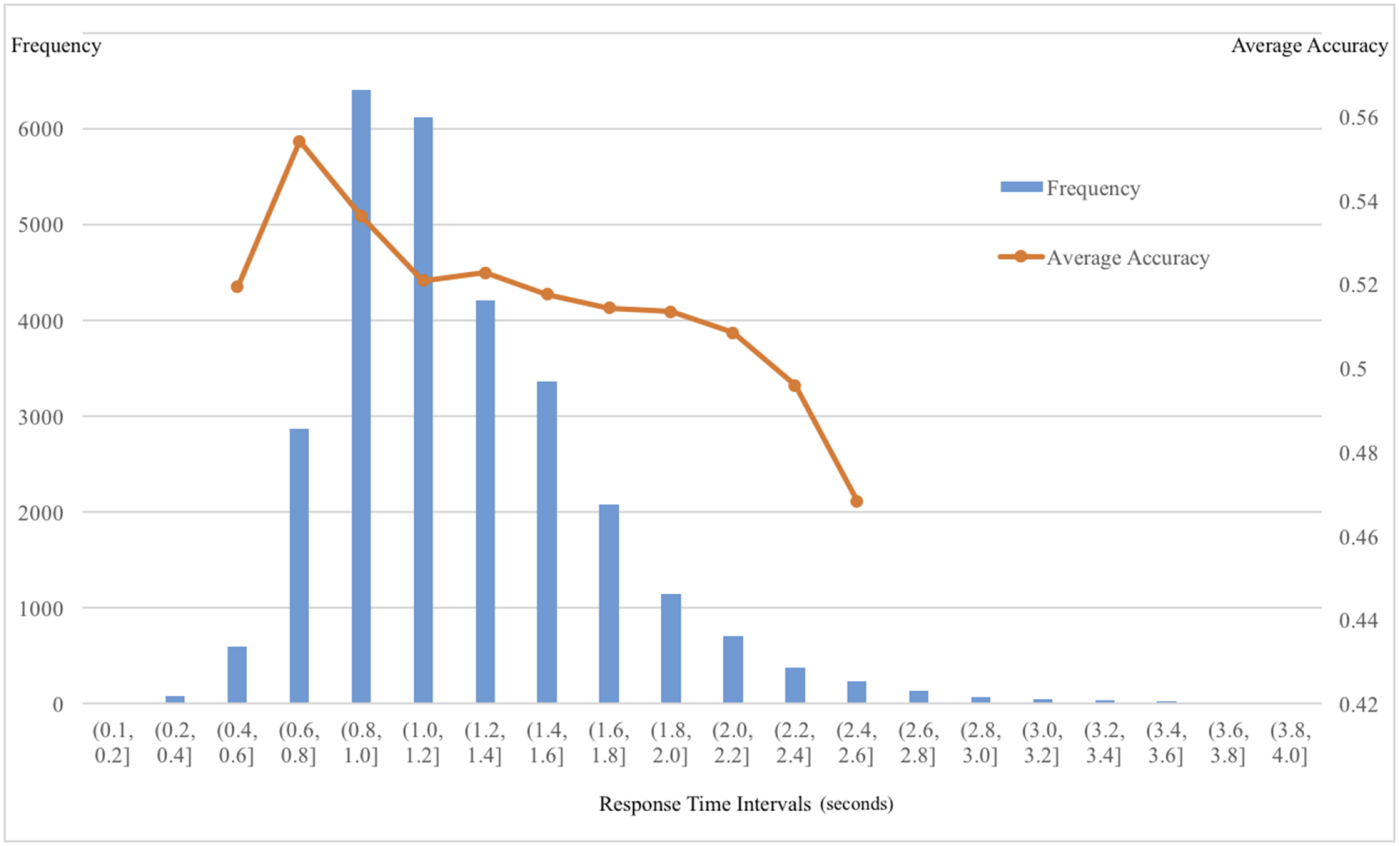
Distribution of response times and average agreement. The histogram represents the distribution of response times over all trials (n = 28540) across all participants, candidate pairs, and traits, excluding missing data, data for recognized candidates, and seven trials with response times less than 100 milliseconds. The line represents the average agreement over trials with response times within the given interval, omitting those for response-time intervals with less than 200 trials.

We investigated how response-times might be related to the above associations between face-based trait inferences and real election outcomes. We had collected a large number of individual observations (nTrial = 28540) across all participants, candidate pairs, and traits, excluding missing data, data for recognized candidates, and seven trials with response times less than 100 milliseconds (the minimum time needed for visual exploration of the faces [3]). The average response time across all trials was 1.23 seconds (SD = 0.44s). In line with prior literature, the distribution of our participants’ response times was similar to the ex-Gaussian distribution (Fig 3). Interestingly, when the percentages of agreement were binned over trials within specific response-time intervals, we found a negative correlation (*rho* = -0.828, 95% CI = [-0.954, -0.453], *p* = 0.002) between response times and agreement percentages (Fig 3).

To further test the effect of response-time on the agreement between face-based trait inferences and election outcomes, we regressed the binary agreements on log-transformed response times in a logit model (Table 6, Model 1). We found that the shorter response times a participant used to make face-based trait inferences, the more likely his/her trait inferences agreed with the real election outcomes. We also controlled for candidates’ cultures (Table 6, Model 2), traits (Table 6, Model 3), participants’ cultures, and all other individual characteristics (Table 6, Model 4). We found a significant negative relation between time and election agreement in all the models, even in those with extensive covariates (Table 6, Model 5; see S2 Table for the complete list of covariates). To account for the correlated errors among responses made by the same participant and those for the same candidate pair, we also clustered the standard errors at individual and image levels (S2 Table, Model 5a). Note that none of the interaction terms had a significant effect, which suggested the negative association between response-time and agreement was invariant of candidates’ cultures, participants’ cultures, and the traits being evaluated.

**Table 6:** The effect of response time on the association between face-based trait inferences and real election outcomes.

	Model 1	Model 2	Model 3	Model 4	Model 5
Log Time	-0.112 **	**-0.093 .**	-0.138 *	-0.215 ***	-0.229 *
	(0.035)	(0.050)	(0.067)	(0.056)	(0.091)
Candidate Culture (1 = Korean)		0.117 ***			0.118 ***
		(0.026)			(0.035)
Candidate Culture * Log Time		**-0.026**			**-0.026**
		(0.069)			(0.073)
Competence			0.135 ***		0.120 **
			(0.037)		(0.037)
Open-mindedness			0.078 *		0.092 *
			(0.037)		(0.037)
Threat			0.074 *		0.078 *
			(0.037)		(0.037)
Competence * Log Time			**0.073**		**0.092**
			(0.096)		(0.105)
Open-mindedness * Log Time			**-0.060**		**-0.065**
			(0.096)		(0.101)
Threat * Log Time			**0.119**		**0.157**
			(0.095)		(0.100)
Participant Culture (1 = Korean)				**-0.048**	**-0.045**
				(0.035)	(0.035)
Participant Culture * Log Time				**0.060**	**0.052**
				(0.050)	(0.050)
Gender (1 = Female)				**0.003**	**0.002**
				(0.027)	(0.027)
Age				**-0.003**	**-0.003**
				(0.003)	(0.003)
Education				0.030 *	0.029 *
				(0.012)	(0.012)
Years in U.S.				0.005 *	0.005 *
				(0.002)	(0.002)
Political Participation: Vote				-0.108 ***	-0.107 ***
				(0.031)	(0.031)
Liberal-Conservative Placement				**0.022 .**	**0.022 .**
				(0.013)	(0.013)
Collectivism Score				0.278 *	0.266 *
				(0.110)	(0.110)
Goodness of Fit: C-index	0.513	0.521	0.520	0.526	0.536

Significant codes: 0 ‘***’ 0.001 ‘**’ 0.01 ‘*’ 0.05 ‘.’ In model 3, corruption was the reference trait. In model 4 and 5, some insignificant individual characteristics were not presented in the table because of limited space. For the complete list of variables, please refer to (S2 Table).

## Discussion

### Summary of results

We reproduced the previously reported finding that candidates perceived as more competent by Caucasian participants were associated with winners in U.S. elections [1]. This finding has been reported in numerous studies, most of which recruited students as participants (e.g., [1–3, 7, 18, 24–26, 38–39, 47–49]). Although there is concern that students may be a poor subject population for studying political decision-making [50], there is also evidence suggesting that subjects of different age groups agree on face-based judgments of competence [12]. In the present study, we recruited participants from the general public and reproduced the basic result in [1]. Moreover, the mean and variance of the percentage of agreement we found were similar to those reported in studies with student samples. Our results strengthen the external validity of the primary finding in this literature.

Contrary to [26], however, we found that inferences of competence, made by participants from both cultures, were about equally and strongly correlated with U.S. and Korean election outcomes. This discrepancy is not likely to be due to the differences in stimuli. We used the same set of Korean candidate images, and followed the same procedures in selecting Caucasian candidate images, as in [26]. Instead, the confounding order effect in [26] and the differences in subject pools between the two studies might have led to the discrepancies in findings. While the ordering of image groups (2 candidate cultures) was randomized in our study for both Caucasian and Korean participants, all Korean participants in [26] evaluated U.S. candidates first. While the Korean participants in our study had lived in the U.S. for at least six months, the Korean participants in [26] were in Korea. It is worth noting that, in our study, how long the Korean participants had lived in the U.S. did not affect the strength of the trait-election association. We found that competence inferences made by “Long-time Koreans” (who had lived in the U.S. for a longer time than the median, 7.5 years) and “New Koreans” (who had lived in the U.S. for a shorter time than the median) were similarly associated with election outcomes (in both countries): (M_LT_ = 53.83%, M_new_ = 53.90%, *d* = -0.07%, t(37) = -0.03, 95% CI = [-5.32%, 5.17%]) for U.S. elections, and (M_LT_ = 54.35%, M_new_ = 51.35%, *d* = 3.00%, t(36) = 1.29, 95% CI = [-1.71%, 7.72%]) for Korean elections. We thus believe that the discrepancy between our findings (of cultural similarity in the association between competence judgments and election outcomes) and the findings of [26] (of cultural differences for the same association) may be traced primarily to order effects in [26].

We found that the specific traits that were most strongly associated with real election outcomes differed between the two countries: while perceived competence (by participants from both cultures) correlated with winning candidates in both countries as just noted, perceived open-mindedness and threat (by participants from both cultures) were associated with winning and losing candidates (respectively) in Korean elections only. One possible explanation for why perceived open-mindedness was associated with Korean election outcomes could be that the Asian transition from more closed to more open societies, and their adaptation to globalization, have encouraged voters to favor more reform-oriented and open-minded political leaders [51]. However, unlike [38, 39], the associations we found between perceived threat and U.S. election outcomes were not significant (though the average percentages of agreement were slightly above chance). This discrepancy is unlikely to be due to differences in stimuli, question wordings, or experimental procedures: our stimuli for Caucasian candidates were randomly selected from the same face database as in [39], our threat evaluation question was worded identically, and our image presentation procedure was also identical to [39]. One possible explanation for the discrepancy might be that student samples (in [39]) and general public samples (in our experiments) differ in how they perceive threat from faces. It will be important for future studies to investigate how student samples and non-student samples might differ in making face-based inferences across a broader profile of negative traits, and how such judgments may depend also on the personality of the viewer.

### Implications for causality

While our study is fundamentally correlational in nature, the findings nonetheless have implications for causal hypotheses. Several studies have investigated whether candidate appearances causally influence voter decisions [30, 44, 52–53]. If voters take visual cues from candidates’ physical appearances when they decide which candidate to vote for, then one would expect that the impact of appearances is greater on those who are exposed to more visual images of the candidates. One of the studies [52] tested this hypothesis on a combined dataset with individual-voter-level data about vote intent, political knowledge, and TV exposure, and candidate-level data about the ratings of their appearances. They found that the effect of candidate appearances was more pronounced among those who had high TV exposure but knew little about the candidates. Another of these studies [53] tested the causal hypothesis by conducting two internet polls in which registered voters intending to vote were randomly assigned to receive standard ballots or ballots with candidate photos. They found that better looking candidates experienced greater success in the ballots with their photos than the standard ballots and that this effect was stronger among low-knowledge voters.

On the other hand, the cultural differences we found provide a new perspective on testing the causal relationship between candidate appearances and election outcomes. If voters evaluate candidates on the traits they value and take visual cues from faces for these evaluations, then one would expect that the specific traits that most strongly associate with election outcomes would differ across cultures because people from different cultures value different traits of their leaders [54, 55]. In our study, almost all cultural effects were driven by the culture of the politicians, not the culture of the participants, which suggests that the differential effect of various traits on election results might arise from how those traits are valued in the respective cultures. To provide causal evidence, future studies could investigate whether open-mindedness and threat have stronger impacts on impression formation and leader evaluation in Korea (or Asian countries) than the U.S. (or Caucasian cultural countries).

### Implications for political behavior

The cultural differences we found also have implications for the study of political behavior. In the classic vote choice model, major considerations were given to social determinants, party identification, and political issues. Studies trying to measure the effects of candidate traits on election outcomes found conflicting results: there was evidence that assessments of candidate traits influenced individual vote choice [56–58], with some arguing that the effects of candidate traits might be mediated by uncertainty and information [59, 60], while others asserted that the net effects of candidate traits might be negligible [61, 62]. Our findings have demonstrated that candidate traits have significant effects on elections and should be included in the classic vote choice model.

### Implications for cognitive psychology

Counter to the usual speed-accuracy trade-off, we found that the shorter the response times a participant took to make face-based trait inferences, the more strongly his/her trait inferences correlated with election outcomes. This finding provides new insights into the higher cognitive processes that might be involved in face-based impression formation. Prior to our study, some [2, 63] have investigated the effects of response-time on the association between trait inferences and election outcomes. By manipulating image exposure time and the response deadline procedure, those studies found that increasing image exposure time after 100 milliseconds did not strengthen the association, and instructing subjects to deliberate in fact weakened the association. However, based on these prior findings, it is not straightforward to conclude that under the same image exposure time and response deadline condition, shorter response times should result in stronger associations, as we found in our study. Moreover, we found negative correlations between response-times and the trait-election associations regardless of candidates’ cultures, participants’ cultures, or the types of traits being evaluated. Faster trait judgments always produced stronger associations.

We suggest two possible explanations for this effect of response-time, which require further investigation. First, the quicker a participant is to make a choice between a pair of candidates, the more likely it is that these two candidates look different, making it easier for the participant to decide which one fits the trait better. On the other hand, taking a longer time to make a choice between two candidates suggests greater uncertainty and difficulty, and therefore the decision tends to be less accurate. Thus, short response times may be correlated with stronger trait-election associations simply because they are derivative to those judgments about pairs of politicians that are also the easiest to make.

A second, and not mutually exclusive, possibility is that evaluating candidates on certain traits by real-world voters may engage mostly “system 1” processes (a type of cognitive process that is quick, automatic, and effortless [64]). That is, when voters actually vote for candidates, they may well be incorporating trait judgments about the candidates into their choices—but such judgments at the time of voting would likely be implicit, automatic processes more aligned with “system 1”. Participants in our experiment, on the other hand, might exhibit a range of processes when making their trait judgments, as reflected in the range of reaction times that they produced. Some of those judgments—the ones with short reaction times—could plausibly be in line with “system 1” processes; whereas, other judgments—the ones with long reaction times—could plausibly reflect “system 2” processes (another distinct type of higher cognitive process that is slow and requires effort [64]), which perhaps even to correct the snap judgments made by system 1. Those trials in the lab with short reaction times might then correspond more closely to the evaluative processing in voters which influences their actual choices (both are “system 1”), and hence show the strongest association with election outcomes. While this second hypothesis is of course very speculative at this stage, it makes predictions about the type of psychological processes that could actually influence voters at the time that they make their election choices, predictions that could be tested in future studies.

### Other mediational effects

It is also interesting that candidate appearances might have stronger effects on some voters than on others. Recent studies [30, 52–53] have investigated how access to information influences the impact of candidate appearances on voter decisions. These studies found that voters with less political information relied more on candidate appearances in their decision-making. We found that inferences made by participants who had lower levels of political participation were more strongly associated with real election outcomes (Table 6). As suggested in our results and [25], individualistic-collectivist orientations might mediate the association between candidate appearances and voter decisions as well. Moreover, political ideology might be yet another contributing factor. One study [29] found that candidates facing conservative electorates benefited from looking more stereotypically Republican, while no relationship between political facial stereotypes and voting was found for liberal electorates. Another study [65] suggested that voters on the right were more responsive to beautiful candidates than voters on the left. In our study, inferences made by more conservative participants were more strongly associated with election outcomes, but this effect of political ideology became insignificant when the correlated errors were adjusted.

Our last point is that some of the images we used are more than a decade old (e.g., some images were of candidates from the 2000 U.S. Senate elections). The development of social media and image processing technology, and the awareness of the association between candidate appearances and election outcomes in the past decade, may have changed the relationship between attribute judgments and election outcomes. It will be important to investigate how the relationships that have been reported to date may change over time.

## Supporting information

S1 Table Subject pool demographic statistics. (PDF)

S2 Table The effect of response time on the association between face-based trait inferences and real election outcomes. (PDF)
