# Supplementary material for: Cultural effects on the association between election outcomes and face-based trait inferences
Source: PLoS One. 2017 Jul 10;12(7):e0180837. doi: 10.1371/journal.pone.0180837 (PMC5507274; doi:10.1371/journal.pone.0180837)
Supplement: S1 Table — (PDF) [file pone.0180837.s001.pdf]

S1 Table. Subject Pool Demographic Statistics.

	Caucasian Participants (N = 40, 20 male)		Korean Participants (N = 40, 20 male)	
	M	SD	M	SD
Age	31	6.93	29	6.42
Education[a]	Associates' Degree	-	Bachelor's Degree	-
Wilson Patterson Scale[b]	0.30	0.13	0.45	0.10
Ideology Placement[c]	2.63	1.37	3.58	1.26
Voted in Election[d]	65.79%	-	32.50%	-
Days/week discussed politics[e]	3.1	1.79	1.23	1.14
Political Campaign[f]	68.42%	-	37.50%	-
Political Knowledge Score[g]	0.70	0.19	0.57	0.20
Individualism Score[h]	0.71	0.14	0.64	0.13
Collectivism Score	0.67	0.17	0.66	0.12

[a]Participants indicated the highest level of education they have completed. The medians were presented in the table.

[b]Participants indicated on a 5-point scale how they felt about each topic in the 16-item Wilson Paterson Scale (Ahn et al., 2014). Response options were strongly agree, agree, uncertain, disagree, and strongly disagree. Responses to conservatism oriented items were scored from 5 to 1; responses to other items were reversed, thus scored from 1 to 5. Scores for the 16 items were averaged for each participant.

[c]Participants indicated their ideology on a 7-point liberal-conservative scale. The question was from American National Election Studies (ANES) Pre 2012. Scores were normalized to [0,1].

[d]Participants indicated whether or not {0,1} they had voted in the last Presidential Election they were eligible to vote, no matter it was in the U.S. or Korea. The question was a variation of Current Population Survey (CPS) Nov 2012, PES1.

[e]Participants indicated during a typical week how many days {0,…,7} they discussed politics with their family or friends. The question was from ANES post 2008.

[f]Participants indicated during the campaign whether they talked to people about who they should vote for or against. The question was from ANES post 2004.

[g]Participants were asked seven questions on their political knowledge. Questions varied in their difficulty and their relevance to U.S. and Korea (e.g., a question asking about United Nations meeting in New York last September and a question asking what political office Ban Ki-moon currently hold.). Scores for each question were weighted on the reversed percentages of participants getting the correct answer, thus the more difficult the higher the weight. Weighted average scores were normalized to [0,1].

[h]Individualism and collectivism scores were calculated based on participants' agreement/disagreement on 16 statements (Singelis, 1995).

References

1. Ahn WY, Kishida KT, Gu X, Lohrenz T, Harvey A, Alford JR, Smith KB, Yaffe G, Hibbing JR, Dayan P, Montague PR. Nonpolitical images evoke neural predictors of political ideology. Current Biology. 2014 Nov 17;24(22):2693-9. doi: 10.1016/j.cub.2014.09.050
2. Singelis TM, Triandis HC, Bhawuk DP, Gelfand MJ. Horizontal and vertical dimensions of individualism and collectivism: A theoretical and measurement refinement. Cross-cultural research. 1995 Aug 1;29(3):240-75. doi: 10.1177/106939719502900302
