# Supplementary material for: Cultural effects on the association between election outcomes and face-based trait inferences
Source: PLoS One. 2017 Jul 10;12(7):e0180837. doi: 10.1371/journal.pone.0180837 (PMC5507274; doi:10.1371/journal.pone.0180837)
Supplement: S2 Table — (PDF) [file pone.0180837.s002.pdf]

S2 Table. The Effect of Response Time on the Association between Face-based Trait Inferences and Real Election Outcomes.

	**Model 1**	**Model 2**	**Model 3**	**Model 4**	**Model 5**	**Model 5a Adj Errors**
Log Time	-0.112 ** (0.035)	-0.093 . (0.050)	-0.138 * (0.067)	-0.215 *** (0.056)	-0.229 * (0.091)	**-0.229 .** (0.137)
Candidate Culture (1 = Korean)		0.117 *** (0.026)			0.118 *** (0.035)	0.118 (0.107)
Candidate Culture * Log Time		-0.026 (0.069)			-0.026 (0.073)	-0.026 (0.105)
Competence			0.135 *** (0.037)		0.120 ** (0.037)	**0.120 *** (0.053)
Open-mindedness			0.078 * (0.037)		0.092 * (0.037)	**0.092 .** (0.048)
Threat			0.074 * (0.037)		0.078 * (0.037)	**0.078 .** (0.042)
Competence * Log Time			0.073 (0.096)		0.092 (0.105)	0.092 (0.129)
Open-mindedness * Log Time			-0.060 (0.096)		-0.065 (0.101)	-0.065 (0.119)
Threat * Log Time			0.119 (0.095)		0.157 (0.100)	0.157 (0.117)
Participant Culture (1 = Korean)				-0.048 (0.035)	-0.045 (0.035)	-0.045 (0.047)
Participant Culture * Log Time				0.060 (0.050)	0.052 (0.050)	0.052 (0.061)
Gender (1 = Female)				0.003 (0.027)	0.002 (0.027)	0.002 (0.030)
Age				-0.003 (0.003)	-0.003 (0.003)	-0.003 (0.003)
Education				0.030 * (0.012)	0.029 * (0.012)	**0.029 *** (0.013)
Years in U.S.				0.005 * (0.002)	0.005 * (0.002)	0.005 (0.003)
Political Participation: Vote				-0.108 *** (0.031)	-0.107 *** (0.031)	**-0.107 **** (0.038)
Political Participation: Talk Politics				0.001 (0.009)	0.001 (0.009)	0.001 (0.009)
Political Participation: Campaign				-0.010 (0.027)	-0.010 (0.027)	-0.010 (0.026)
Wilson Patterson Scale				0.142 (0.152)	0.149 (0.152)	0.149 (0.192)
Liberal-Conservative Placement				0.022 . (0.013)	0.022 . (0.013)	0.022 (0.020)
Political Knowledge Score				-0.117 . (0.070)	-0.110 (0.070)	-0.110 (0.099)
Individualism Score				-0.010 (0.105)	-0.012 (0.105)	-0.012 (0.112)
Collectivism Score				0.278 * (0.110)	0.266 * (0.110)	**0.266 .** (0.139)
Goodness of Fit: C-index	0.513	0.521	0.520	0.526	0.536	0.536

Significant codes: 0 '***' 0.001 '**' 0.01 '*' 0.05 '.'
